# Global transcription profiling reveals differential responses to chronic nitrogen stress and putative nitrogen regulatory components in Arabidopsis

**DOI:** 10.1186/1471-2164-8-281

**Published:** 2007-08-16

**Authors:** Yong-Mei Bi, Rong-Lin Wang, Tong Zhu, Steven J Rothstein

**Affiliations:** 1 Department of Molecular and Cellular Biology, University of Guelph, Guelph, Ontario, N1G 2W1, Canada; 2 Ecological Exposure Research Division, National Exposure Research Lab, US EPA, 26 W. Martin Luther King Dr., Cincinnati, OH 45268, USA; 3 Syngenta Biotechnology Inc., 3054 Cornwallis Road, Research Triangle Park, North Carolina, 27709, USA

## Abstract

**Background:**

A large quantity of nitrogen (N) fertilizer is used for crop production to achieve high yields at a significant economic and environmental cost. Efforts have been directed to understanding the molecular basis of plant responses to N and identifying N-responsive genes in order to manipulate their expression, thus enabling plants to use N more efficiently. No studies have yet delineated these responses at the transcriptional level when plants are grown under chronic N stress and the understanding of regulatory elements involved in N response is very limited.

**Results:**

To further our understanding of the response of plants to varying N levels, a growth system was developed where N was the growth-limiting factor. An Arabidopsis whole genome microarray was used to evaluate global gene expression under different N conditions. Differentially expressed genes under mild or severe chronic N stress were identified. Mild N stress triggered only a small set of genes significantly different at the transcriptional level, which are largely involved in various stress responses. Plant responses were much more pronounced under severe N stress, involving a large number of genes in many different biological processes. Differentially expressed genes were also identified in response to short- and long-term N availability increases. Putative N regulatory elements were determined along with several previously known motifs involved in the responses to N and carbon availability as well as plant stress.

**Conclusion:**

Differentially expressed genes identified provide additional insights into the coordination of the complex N responses of plants and the components of the N response mechanism. Putative N regulatory elements were identified to reveal possible new components of the regulatory network for plant N responses. A better understanding of the complex regulatory network for plant N responses will help lead to strategies to improve N use efficiency.

## Background

Nitrogen (N) is the most important inorganic nutrient for plant growth [1]. It affects many aspects of plant growth and development, such as N and carbon (C) allocation, root branching, leaf growth and flowering time [2-5]. The production of high-yielding crops is associated with the application of a large quantity of fertilizers at a substantial cost [6], and N pollution is becoming a threat to global ecosystems [7,8]. Efforts have been directed to understanding the molecular basis of plant responses to N and to identifying N-responsive genes in order to manipulate their expression and enable plants to use N more efficiently [9].

Nitrate is the major source of N in agricultural soils [10]. It serves as a nutrient and as a signal [11,12]. As a nutrient, it is taken up by the low and high affinity nitrate transporter gene family members (NRT1 and NRT2), reduced to nitrite by nitrate reductase (NR), and to ammonium by nitrite reductase (NiR). Ammonium is then incorporated into amino acids, catalyzed primarily by glutamine synthetase (GS) and glutamate synthase (GOGAT) [11-14]. As a signal, it can induce the expression of a number of genes including *NRT1*, *NRT2*, *NR *and *NiR *[4,12,15,16]. The expression of the ammonium assimilatory genes, *GS *and *GOGAT*, are also induced by the addition of nitrate [12,13], as are genes involved in sugar metabolism and other metabolic pathways [17]. In addition to these metabolic genes, expression of some regulatory genes is also affected by N levels. For example, the Arabidopsis MADS-box gene *ANR1*, which regulates lateral root development, responds to N, but not potassium or phosphate [3].

There have been several studies of plant N-responses based on microarray gene expression profiling. Wang et al [18] studied the response of seedlings grown on ammonium to the addition of low or high levels of nitrate. They used the Arabidopsis GEM1 microarrays, which contained 7942 cDNA clones corresponding to 5524 unique genes, and identified 25 and 49 N-responsive genes to low or high nitrate induction, respectively [18]. Subsequently, Wang et al [19] used the Arabidopsis whole-genome Affymetrix ATH1 microarray containing 22,626 genes, to study the addition of the low level of nitrate to discover more N-responsive genes. Scheible et al [20] also used the ATH1 microarray combined with real-time RT-PCR of > 1,400 transcription factor genes to identify genes affected by N-deprivation or N-induction after 30 min or 3 hr from N-starved seedlings. Since C and N metabolism are very closely linked and tightly regulated [21,22], Price et al [23] used the ATH1 microarray to identify the individual contributions of nitrogen, sugar, and nitrogen plus sugar on global gene expression. Recently, Lian et al reported expression profiles of 10,422 genes at an early stage of low N stress in rice seedling [24].

So far, these studies have provided valuable insights into N response and its linkage to other biological pathways. However, no studies have yet delineated the responses at the transcriptional level when plants are grown under chronic N stress. Earlier reports involved investigations of transient changes in gene expression when nitrate is added to nitrate-starved seedlings [18-20,23]. As such, many questions still remain unanswered. For example, it is not clear how mature plants would respond to different degrees of chronic N stress. A growth system was developed where N was the growth-limiting factor, so the transcriptional changes of genes that were most affected by different degrees of N limitation could be investigated. Additionally, the global transient changes during N induction were examined by transferring mature plants from low N to high N instead of using N-starved seedlings. In addition, putative regulatory elements involved in N response were also identified as an important first step toward understanding N regulatory networks. Previous progress in this area is limited to nitrate induction from deletion analysis of *NR *or *NiR *promoters [25-29], and a study on the interaction between C and N signaling [30].

## Results and discussion

### Developing defined nitrogen growth conditions for expression profiling

**Figure 1:**
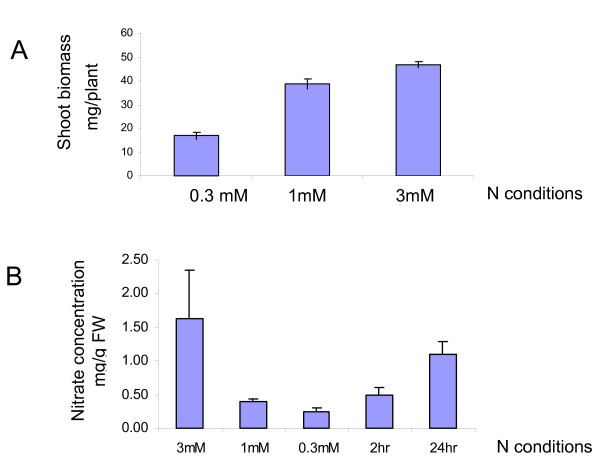
Three-week-old wild type Arabidopsis plants under different N conditions. A: Shoot biomass. Average of shoot biomass (mg/plant) of 6 to 8 three-week-old wild type Arabidopsis plants under the N-sufficient (3 mM), mild (1 mM) and severe (0.3 mM) N-limiting conditions are presented, as well as sd. B: Nitrate levels: Average of nitrate levels (mg/g fresh weight) of 3 biological samples under different N conditions (each sample from a pool of 3 plants) are presented, as well as sd.

To apply chronic N stress, it is important to develop a defined nitrogen growth condition. It is difficult to maintain a constant N level in a soil system due to the different size and affinity of soil particles for nutrients. A "pure" hydroponic system could control the N level well but the root system is not supported by a substrate and it is bathed directly in the nutrient solution with poor aeration. To overcome those shortcomings, a hydroponic system using rockwool as the growing substrate was adopted [31]. The key quality of high grade rockwool is uniform wetting. It provides a buffering reservoir of nutrient solution in the root zone, while maintaining an adequate volume of air (oxygen). A detailed description of the system is provided in the Methods section. Wild-type Arabidopsis (Columbia ecotype) plants at 3 weeks of age were evaluated for shoot biomass under different N conditions, ranging from 0.1 mM to 10 mM. Under these N conditions, 3 mM nitrate was found to give maximal growth (data not shown). For subsequent experiments, 3 mM nitrate was used to produce the N-sufficient condition, 1 mM nitrate to produce the mild N-limiting condition, under which plant growth measured by shoot biomass was reduced to approximately 80% of that at 3 mM nitrate, and 0.3 mM nitrate to produce the severe N-limiting condition, under which plant growth measured by shoot biomass was further reduced to approximately 35% of that at 3 mM nitrate (Figure 1a). Under these conditions, the nitrate levels in the leaves ranged from 0.24 mg/g FW to 1.64 mg/g FW (Figure 1b). Plants have a nitrate transport system that could have a very high affinity for nitrate [32] and provide a certain capacity for nitrate absorption at low external nitrate concentrations. However, in our system, the amount of nitrate in one rockwool cube that was available to support the growth of one plant was apparently very limited under the low N conditions. The consequence was the obvious difference in growth under these N conditions. Apart from reduced shoot biomass due to N deficiency, a reduction in chlorophyll level was also observed (data not shown).

### Identification of differentially expressed genes by expression profiling

Shoots of 3-week-old wild-type plants grown under the N-sufficient condition (3 mM nitrate), the mild N-limiting condition (1 mM nitrate) and the severe N-limiting condition (0.3 mM nitrate) were harvested for the profiling experiment to compare the baseline gene expression levels under different, but stable N conditions. Since it is known that the expression levels of nitrate assimilation genes are high early in the day and low late in the day, all samples were taken at the middle of the day to minimize diurnal changes in C and N metabolism [33]. Each condition had three biological replicates. Also, at 2 hrs or 24 hrs before the harvest, some of the plants grown under the severe N-limiting condition (0.3 mM nitrate) were transferred to the N-sufficient condition (3 mM nitrate), and then collected at the same time as those grown under different stable N conditions to assess gene expression changes after 2 hr or 24 hr N induction. Nitrate levels in the leaves increased from 0.24 mg/g FW to 0.49 mg/g FW 2 hr after addition of higher levels of nitrate and to 1.09 mg/g FW after 24 hr (Figure 1b). Again, three biological repeats were collected for each time point. RNA was extracted and hybridized to a custom designed Arabidopsis GeneChip^® ^whole genome microarray. This microarray contains 26,412 probe sets representing 26,367 Arabidopsis known and predicted genes. On average, each gene contains 15 perfect match probes, selected from the 3' end of 133,397 exons [34].

Significance analysis of microarray (SAM; academic version 2.23B) [35] was used to identify genes differentially expressed between the following: (1) the N-sufficient to the mild N-limiting samples (3 mM N to 1 mM N); (2) the N-sufficient to the severe N-limiting samples (3 mM N to 0.3 mM N); (3) severe N (0.3 mM N) to the 2 hr N induction samples; and (4) severe N (0.3 mM N) to the 24 hr N induction samples. Different numbers of significant genes could be obtained depending on the SAM median false discovery rate (FDR). The final number of significant genes of the following analyses was based on a median FDR of 0% and a minimum of a 2-fold expression difference (Table 1). To confirm the results of the microarray analysis, the expression of 14 significant genes (7 up-regulated and 7 down-regulated) was tested by semi-quantitative RT-PCR and found to be consistent with the microarray data [see Additional file 1].

**Table 1:** Significant gene numbers at different cutoff levels

	**FDR 0.5%**	**FDR 0%**	**FDR 0% minimum 2 fold**
Sufficient N vs. mild N	157	120	52 (51^a^, 1^b^)
Sufficient N vs. severe N	2176	461	461 (271^a^, 190^b^)
Severe N vs. 2 hr induction	146	90	90 (77^a^, 13^b^)
Severe N vs. 24 hr induction	2523	905	837 (481^a^, 356^b^)

Significance analysis of microarray (SAM; academic version 2.23B) was used to identify differentially expressed genes. Different number of significant genes could be obtained depending on the SAM median false discovery rate (FDR) and a minimum fold change. ^a ^number of genes up-regulated; ^b ^number of genes down-regulated

### Functional classification of significant genes under mild and severe chronic N stress

Although plant growth measured by shoot biomass was reduced by 20% under mild N stress, the baseline expression levels of most genes in the genome remained similar. Only 52 genes were identified with expression levels significantly different, of which 51 were induced and one suppressed in the mild N-limiting condition compared to the N-sufficient condition. However, when the degree of this chronic N stress increased, these numbers increased dramatically. A total of 461 genes showed significant response, of which 271 genes were up-regulated and 190 genes down-regulated under the severe N-limiting condition, when plant growth was reduced by 65% measured by shoot biomass. Severe N stress introduced a much greater transcriptional changes compared to mild N stress. This result is similar to the microarray study on plants under different degrees of phosphate deficiency [36]. In that case, 72 genes were induced and four genes were suppressed during short-term Pi deprivation, and the numbers increased to 291 genes induced and 34 genes suppressed during medium-term Pi starvation, and further increased to 501 genes induced and 231 genes suppressed during long-term deficiency [36].

The entire gene lists under the mild and severe N-limiting conditions are provided in Additional file 2 and 3 respectively, together with their functional and pathway assignments. Functional assignments are defined by Gene Ontology (GO) terms [37], which provide broad functional classifications for genes and gene products representing their corresponding biological processes, molecular function, and cellular localization. Pathway assignments are derived from the Kyoto Encyclopedia of Genes and Genomes (KEGG) [38]. Gene ontology on the Arabidopsis Information Resource website [39], and a browser-based functional classification program [40] were also consulted.

Under the mild N-limiting condition when plant growth was slightly reduced, none of the genes directly involved in nitrate assimilation showed significant changes in expression (Table 2). However, when plants were grown under the severe N-limiting condition, many primary metabolism genes including those involved in N assimilation were significantly down-regulated. In terms of the nitrate assimilatory genes, the baseline expressions of *NR1, NR2 *and *NiR *were clearly correlated with the three N levels – the lower the N concentration, the lower the baseline expression (Table 2). This indicates that the degree of the reduction of N assimilation gene expression is correlated to the amount of N available. As for the ammonium assimilatory genes, *GS1-1 *and *GS1-4*, the opposite trend of the nitrate assimilation genes (*NR1, NR2 *and *NiR*) was observed. *GS1-1 *and *GS1-4 *were up-regulated under severe N stress (Table 2). GS has a very high affinity for ammonia and catalyzes the ATP-dependent conversion of glutamate into glutamine by incorporating a molecule of ammonia. The individual isoenzymes of GS and GOGAT have been proposed to play roles in three major ammonium assimilation processes: (1) primary nitrogen assimilation; (2) re-assimilation of photorespiratory ammonia; and (3) re-assimilation of "recycled" nitrogen [41]. Since primary metabolism such as nitrogen assimilation, photosynthesis and photorespiration is repressed under severe N stress, it is likely that the increased expression of cytosolic GS1 is related to the re-assimilation of ammonia released from protein degradation which usually occurs under N deficiency. With regard to the N uptake genes, different trends were observed among members of the low affinity nitrate transporter gene family *NRT1. NRT1.1 *baseline expression was higher, while *NRT1.3 *and *NRT1.4 *baselines expression were lower under N stress (Table 2), suggesting a lack of functional redundancy among family members. In addition to the N assimilation genes, those involved in the nitrate storage were deeply repressed by N deficiency. An anion channel protein gene, which was recently demonstrated to mediate nitrate accumulation in plant vacuoles [42], was down-regulated almost 7-fold under severe N shortage while no significant difference in expression was observed under mild N limitation (Table 3).

**Table 2:** Expression of nitrate assimilation genes under different N conditions

Genes		Locus	Fold limiting	Fold stress	Fold 2 hr	Fold 24 hr
**Nitrate transport**						
*Low-affinity nitrate transporter*	*NRT1.1*	*At1g12110*	1.3	1.8	1.8	-1.3
	*NRT1.2*	*At1g69850*	-	-	-	-
	*NRT1.3*	*At3g21670*	-1.1	-2.1	1.0	1.6
	*NRT1.4*	*At2g26690*	-1.1	-2.1	1.2	2.4*
*High-affinity nitrate transporter*	*NRT2.1*	*At1g08090*	-	-	-	-
	*NRT2.2*	*At1g08100*	-	-	-	-
	*NRT2.3*	*At5g60780*	-	-	-	-
	*NRT2.4*	*At5g60770*	-	-	-	-
	*NRT2.5*	*At1g12940*	-	-	-	-
	*NRT2.6*	*At3g45060*	-	-	-	-
	*NRT2.7*	*At5g14570*	-	-	-	-
**Nitrate reduction**						
*Nitrate reductase 1 (NR1)*		*At1g77760*	-1.2	-1.9	11.1*	1.5
*Nitrate reductase 2 (NR2)*		*At1g37130*	-1.2	-3.3*	8.1*	2.9*
*Nitrite reductase (NiR)*		*At2g15620*	-1.2	-4.4*	15.1*	3.2*
*urophorphyrin III methylase (UPM1)*		*At5g40850*	1.0	-1.5	10.1*	1.5
*Fd NADP (+) reductase (FNR)*		*At4g05390*	1.3	1.2	3.6*	1.0
		*At1g30510*	1.1	1.1	3.1*	1.0
		*At4g32360*	-	-	-	-
		*At5g66190*	-	-	-	-
		*At1g20020*	-	-	-	-
		*At5g66810*	-	-	-	-
**Ammonia assimilation**						
*Glutamine synthetase*	*GS1-1*	*At5g37600*	1.7	4.7*	-1.6	-3.4*
	*GS1-2*	*At1g66200*	-	-	-	-
	*GS1-3*	*At3g17820*	-	-	-	-
	*GS1-4*	*At5g16570*	1.7	6.9*	-2.0	-3.7*
	*GS1-5*	*At1g48470*	-	-	-	-
*Glutamate synthetase*	*GS2*	*At5g35630*	-	-	-	-
	*GOGAT1*	*At5g04140*	-	-	-	-
	*GOGAT2*	*At2g41220*	-	-	-	-
	*GOGAT3*	*At5g53460*	-	-	-	-
*Asparagines synthetase*	*AS1*	*At3g47340*	-	-	-	-
	*AS2*	*At5g65010*	-1.3	-3.2*	7.6*	2.9*
	*AS3*	*At5g10240*	-	-	-	-

Fold changes under different N conditions were presented: sufficient N vs. limiting N (fold, limiting); sufficient N vs. stress N (fold, stress); stress N vs. 2 hr induction (fold, 2 hr); stress N vs. 24 hr induction (fold, 24 hr). Negative number means gene expression down-regulated in said condition, and positive number means gene expression up-regulated in said condition. *significant from our SAM analysis;

---- no fluctuation under all N conditions

**Table 3:** Expression of selected significant genes under different N conditions

Functional classification	Locus	Gene identification	Fold limiting	Fold stress	Fold 2 hr	Fold 24 hr
nitrate accumulation	*At5g40890*	anion channel protein	ns	-6.8	2.9	6.0
photosynthesis	*At1g03600*	photosystem II family protein	ns	-2.9	ns	2.3
	*At4g28660*	photosystem II protein W	ns	-2.7	ns	3.2
pentose-phosphate pathway	*At5g35790*	glucose-6-phosphate 1-dehydrogenase	ns	-2.6	ns	2.3
	*At1g24280*	glucose-6-phosphate 1-dehydrogenase	ns	ns	5.2	ns
starch synthesis	*At4g39210*	ADP-glucose pyrophosphorylase	ns	3.9	ns	-4.1
	*At2g21590*	ADP-glucose pyrophosphorylase	ns	2.8	ns	-3.1
chlorophyll synthesis	*At1g03630*	protochlorophyllide reductase	ns	-2.9	ns	2.8
	*At4g27440*	protochlorophyllide reductase	ns	-2.9	ns	2.3
	*At5g08280*	hydroxymethylbilane synthase	ns	-2.6	ns	2.7
protein degradation	*At5g45890*	senescence-specific cysteine protease SAG12	ns	7.7	ns	-4.3
nitrogen detoxification	*At5g22300*	nitrilase 4	2.8	11.5	ns	-4.4
anthocyanin synthesis	*At4g22880*	leucoanthocyanidin dioxygenase	2.1	14.4	ns	-7.8
	*At5g42800*	dihydroflavonol 4-reductase	3.3	18.7	ns	-8.3
	*At5g13930*	chalcone synthase	ns	4.1	ns	-3.4
	*At1g56650*	Myb transcription factor PAP1	ns	6.3	-3.0	-6.3
	*At1g66390*	Myb transcription factor PAP2	3.5	26.6	-6.7	-16.7
phenylpropanoid synthesis	*At2g37040*	phenylalanine ammonia lyase	ns	4.1	ns	-3.2
response to cytokinin	*At3g63110*	cytokinin synthase	ns	ns	3.6	ns
	*At3g48100*	transcription repressor	ns	-4.3	3.7	3.6
	*At2g18300*	transcription factor	ns	-8.4	7.9	6.8
transcription	*At1g30500*	CCAAT transcription factor	ns	4.3	ns	-4.3
	*At2g34720*	CCAAT transcription factor	ns	3.4	ns	-3.0
	*At3g05690*	CCAAT transcription factor	ns	3.8	ns	ns
	*At5g47220*	ATERF2 transcription factor	2.0	2.4	ns	ns

Fold changes under different N conditions were presented: sufficient N vs. limiting N (fold, limiting); sufficient N vs. stress N (fold, stress); stress N vs. 2 hr induction (fold, 2 hr); stress N vs. 24 hr induction (fold, 24 hr). Negative number means gene expression down-regulated in said condition, and positive number means gene expression up-regulated in said condition. ns – not significant from our SAM analysis

N stress led to marked changes in the expression of genes involved in carbon metabolism. Genes involved in photosynthesis, including those that code for the photosystem II family protein, the oxygen evolving enhancer 3 PsbQ family protein, the PSI type II chlorophyll a b-binding protein, and the photosystem II reaction centre W PsbW family protein were significantly down-regulated under the severe N-limiting condition, but not the mild N-limiting condition (Table 3). In addition, genes involved in the oxidative pentose-phosphate pathway that provide reducing power (NADPH) and pentose phosphates were down-regulated, including the gene coding for glucose-6-phosphate 1-dehydrogenase, which is the rate-limiting step in this process (Table 3). On the other hand, genes involved in the accumulation of starch, including ADP-glucose pyrophosphorylase which catalyzes the first, rate-limiting step in starch biosynthesis, were significantly up-regulated under severe N stress (Table 3).

N stress induces profound changes to growth and development. In order to adapt to the growth arrest due to severe N deficiency, many ribosomal genes involved in protein biosynthesis were down-regulated, including genes coding for structural constituents of ribosome and translation elongation factors [see Additional file 3]. At the same time, genes involved in protein degradation were up-regulated under severe N stress, such as the Cys peptidase (SAG12) (Table 3). It is believed that during nutrient deficiency, plants transport a series of cytosolic proteins into the vacuole, where various proteases are located, to be degraded into amino acids and exported from senescing tissues for cell reuse.

As expected, there were marked changes in the expression of genes involved in hormone synthesis and sensing since they largely control plant growth. Auxin stimulates cell devision and elongation, and many auxin-induced genes were down-regulated under severe N stress [see Additional file 3]. Cytokinin regulates cell proliferation and differentiation, and many cytokinin-responsive genes were down-regulated under severe N stress, including two transcription regulators (*At3g48100, At2g18300*) (Table 3).

Leaf yellowing is one of the typical responses plants have when N deficiency occurs [43]. While chlorophyll levels were slightly reduced (~5% reduction) under the mild N- limiting condition, none of the chlorophyll biosynthesis genes changed appreciably at the transcriptional level, indicating a disconnection between transcriptional responses and phenotypic changes. Under the severe N-limiting condition, the chlorophyll levels were significantly reduced (~30% reduction). Genes involved in chlorophyll metabolism were down-regulated, including protochlorophyllide reductase, protochlorophyllide reductase and hydroxymethylbilane synthase (Table 3).

N stress induced a number of plant stress responses. Many genes involved in various stress responses were up-regulated, including some peroxidase genes and glutathioneS-transfeerase (*GST*) genes under both N-limiting conditions [see Additional file 2 and 3]. Toxic nitrogen compounds are usually generated under various stresses. Nitrilase 4, of which the purified enzyme has been shown to be involved in the nitrogen compound detoxification pathway, was up-regulated around 3-fold under mild N stress, but up-regulated over 11-fold under severe N stress (Table 3).

The presence of the purple flavonoid anthocyanin is an indicator of stress [44], and N deficiency may lead to increased anthocyanin synthesis [43]. Plants were obviously purple in color under the severe N-limiting condition (not shown). Genes involved in anthocyanin synthesis, such as leucoanthocyanidin dioxygenase and dihydroflavonol reductase, were up-regulated just over 2-fold under mild N stress, but increased to about 14- and 18-fold under severe N stress (Table 3). Chalcone synthase, which participates in the biosynthetic pathway for all flavonoids and is required for the accumulation of anthocyanins, was up-regulated only under severe N stress (Table 3). The expression of the Myb transcription factor *PAP1 *was increased about 6-fold under severe N stress, and the expression of the Myb transcription factor *PAP2 *was increased around 3-fold under the mild N-limiting condition, but dramatically increased to over 26-fold under the severe N-limiting condition (Table 3). The two Myb genes have been shown to be able to regulate flavonoid and anthocyanin biosynthesis and over-expression of the Myb genes resulted in an enhanced accumulation of lignin and flavonoids, including various anthocyanins that produce purple color [45]. It is clear that the accumulation of anthocyanins is closely correlated with the level of N stress, and *PAP1 *and *PAP2 *are involved in the regulation of this process. However, it is unclear how specific this regulation is for N stress response.

As N deficiency responses are known to be regulated at the transcriptional level, transcriptional factor genes were looked up from the entire gene lists [see Additional file 2 and 3]. Three transcription factors genes were induced under mild N limitation, including a WRKY transcription factor (*At1g80840*), an ethylene responsive element binding factor 2 (ATERF-2, *At5g47220*) and the aforementioned Myb transcription factor (*PAP2*) gene [see Additional file 2]. Plant proteins containing WRKY or ATERF domains are known regulators of abiotic and biotic stress responses [46,47]. Detailed characterization of the WRKY gene (*At1g80840*) is lacking, but the ATERF-2 gene (*At5g47220*) is known to be regulated by other abiotic stress conditions [48]. Thirty-nine transcription factor genes were significantly induced or repressed under severe N stress, including the Myb factors *PAP1 *and *PAP2*, the two cytokinin-responsive transcription regulators (*At3g48100, At2g18300*) mentioned earlier, and three genes (*At1g30500, At2g34720 *and *At3g05690*) coding for CCAAT binding factor complex (Table 3). While members in this CCAAT family are known to be involved in developing the tolerance of plants to various abiotic stress conditions [49-51], mechanistic details about these CCAAT genes and their possible specific involvement in N stress tolerance need further investigation. Some Myb transcription factors were down-regulated under severe N stress, including two Myb factors (*At1g25550 *and *At1g68670*), and some were up-regulated (*PAP1 *and *PAP2*), indicating the divergent function of individual members within this transcription factor gene family.

Overall, mild N stress triggered only a small set of genes to be expressed significantly differently. These genes are largely involved in various stress responses, including genes involved in anthocyanin biosynthesis. There is a possible disconnect between transcriptional responses and phenotypic changes, as no genes specifically involved in growth arrest or chlorophyll synthesis have significantly altered expression levels. Thus, it appears that there was a compensatory response to this limited level of N stress. Under severe N stress, however, plant responses were much more pronounced. Table 4 summarizes some of the biological processes with significant genes over-represented in the two N stress responses (P < 0.01). Among the up-regulated genes under mild N stress, 16 genes, or 40% of the significant gene list (excluding genes of unknown functions), belong to the "response to abiotic stimulus" process according to GO. Genome-wide, there are 1091 member genes in this process, or 6% of the genome. Apparently, mild N stress invoked a significant response from abiotic stimulus related pathways (P-value = 4.2E-10). Fourteen genes, or 35% of the list, are responsive to different type of stress, including 5 of those responsive to oxidative stress. Six genes, or 15% of the list, are involved in secondary metabolism, including two for flavonoid biosynthesis and two for anthocyanin biosynthesis. Under severe N stress, up-regulated genes are still over-represented by those involved in the response to abiotic stimulus and other types of stress as well as secondary metabolism. However, the relative percentage of these "stress genes" actually decreased when many more genes responded transcriptionally to severe N stress and many of them are involved in other processes, such as the response to abscisic acid stimulus, phenylpropanoid biosynthesis, senescence and starch biosynthesis (Table 4). Genes involved in primary metabolism had a preponderance of down-regulated genes under severe N stress. Fifty-eight genes, or 43% of the list, belong to that category, including 7 involved in nitrogen compound metabolism and 4 in the main pathways of carbohydrate metabolism (Table 4). Twenty-eight biosynthetic genes, or about 20% of the list, are down-regulated, with 17 of these involved in protein biosynthesis. Down-regulated genes are also involved in photosynthesis, the response to auxin and cytokinin and the generation of precursor metabolites and energy (Table 4). The number of genes with significantly altered expression in each of these processes and their degrees of over-representation is noted in Table 4.

**Table 4:** Biological processes with significant genes over-represented under mild or severe chromic N stress

Biological Process	No. in Genome	% in Genome	Under mild N stress			Under severe N stress		
			
			No. in List	% in List	P-value	No. in List	% in List	P-value
**Up-regulated**								
response to abiotic stimulus	1091	6.0	16	40	4.2E-10	39	19.3	7.3E-11
response to stress	945	5.2	14	35	6.4E-09	23	11.4	0.0004
secondary metabolism	249	1.4	6	15	1.6E-05	15	7.4	1.3E-07
response to oxidative stress	140	0.8	5	12.5	1.4E-05	8	4.0	0.0002
flavonoid biosynthesis	30	0.2	2	5	0.002	6	3.0	8.4E-07
anthocyanin biosynthesis	8	0.04	2	5	0.007	3	1.5	7.3E-05
phenylpropanoid biosynthesis	79	0.4				9	4.5	2.3E-07
response to abscisic acid stimulus	113	0.6				9	4.5	4.8E-06
organ senescence	12	0.07				3	1.5	0.0003
starch biosynthesis	7	0.04				2	1.0	0.002
**Down-regulated**								
primary metabolism	5170	28.5				58	43.0	0.0002
biosynthesis	1269	7.0				28	20.7	1.6E-07
protein biosynthesis	500	2.8				17	12.6	1.9E-07
response to auxin stimulus	191	1.1				10	7.4	1.7E-06
generation of precursor metabolites and energy	451	2.5				10	7.4	0.002
nitrogen compound metabolism	276	1.5				7	5.2	0.005
response to cytokinin stimulus	31	0.2				5	3.7	3.1E-06
main pathways of carbohydrate metabolism	95	0.5				4	3.0	0.006
photosynthesis	44	0.2				3	2.2	0.004

The gene list was imported into GeneSpring. The number of genes in each biological process either in a whole genome (No. in Genome), or in the list (No. in list), as well as their corresponding percentage and p-value was generated by GeneSpring.

Around 50% of the genes with significantly altered expression from the mild N-limiting condition overlap with the genes from the severe N-limiting condition. The overlapping gene list is provided in Additional file 4. Many of these genes are involved in the response to abiotic or biotic stress, such as peroxidases, *GST *genes and genes involved in anthocyanin synthesis, pointing to possible interactions between general stress and N stress. Two transcription factors *PAP2 *and ATERF2 (*At5g47220*) are present in both lists. Some of the overlapping genes such as *PAP2 *responded much more strongly at the transcript level under severe N stress, while others such as the ATERF2 gene responded at a similar level under both conditions (Table 3). Therefore, plants might have a unique scaled response system for different degrees of N stress by a gradual activation of genes from mild to severe N stress.

### Functional classification of significant genes after short- and long-term N availability increase

Three-week-old Arabidopsis plants grown under severe N stress were transferred to the sufficient N condition and harvested after 2 and 24 hrs. After 2 hrs of increased N availability, a total of 77 genes were significantly up-regulated and 13 genes were significantly down-regulated. Twenty-four hrs of increased N availability invoked a greater plant response as the expression levels of 481 genes were significantly up-regulated and 356 were significantly down-regulated. The significant gene lists are provided in Additional file 5 and 6 respectively, together with their functional and pathway assignments. The short-term N availability increase triggered a rapid response of many genes directly involved in plant growth and development. The nitrate assimilatory genes, *NR1, NR2 *and *NiR*, were up-regulated significantly after a 2 hr N induction, followed by a drop after 24 hr (Table 2), a pattern consistent with previous reports. The *UPM1 *gene, which encodes an enzyme that makes a cofactor for NiR, had a very similar trend as *NiR *(Table 2). Two *FNR *genes, involved in supplying reductant to NiR, also had a transient increase (Table 2). With regard to the N uptake genes, the low affinity nitrate transporter gene *NRT1.1 *showed some increase after 2 hr N induction, while *NRT1.4 *showed significant induction after 24 hr (Table 2), suggesting a different role played by individual transporters in N response. Surprisingly, none of the seven *NRT2 *members showed significant change (Table 2). High affinity nitrate transporters (eg. *NRT2.1*) were induced significantly from N-starved seedlings [19,20], but not in our experiments, possibly due to the fact that plants in this study were grown at a constant low level of nitrate, rather than being N-starved prior to the addition of a high level of nitrate. The expression of GS and GOGAT, the principle players in ammonium assimilation, didn't change significantly after 2 hr. *GS1-1 *and *GS1-4 *were repressed after the 24 hr induction (Table 2), which is similar to the previous report [20]. Asparagine synthetase (*AS*) plays an important role in amino acid synthesis [41]. Among the three *AS *genes, *AS2 *(*At5g65010*) showed a very similar pattern to the nitrate reduction genes (*NR1, NR2 *and *NiR*) (Table 2). Genes involved in nitrate storage were significantly up-regulated. The anion channel protein gene, mediating nitrate accumulation in plant vacuoles [42], was up-regulated around 3-fold after 2 hr of N addition and continued to increase to 6-fold after 24 hr (Table 3).

Many genes involved in carbon metabolism were up-regulated with increased N availability. Sugar transporter genes were up-regulated after 2 hr, together with some amino acid transporters [see Additional file 5]. The expression of the genes coding for the rate-limiting step of the oxidative pentose-phosphate pathway glucose-6-phosphate 1-dehydrogenase was induced. However, the two genes coding for the two isozymes showed a different pattern with one being induced only after 2 hrs and the other only after 24 hrs of increased N availability (Table 3). Genes involved in photosynthesis (photosystem II family protein and photosystem II reaction centre W PsbW family protein) were significantly up-regulated, and genes involved in the accumulation of starch (ADP-glucose pyrophosphorylase) were down-regulated only in the 24 hr induction samples (Table 3). Phytohormone genes coding for cell growth and expansion were induced, such as adenylate isopentenyl transferase 3 (cytokinin synthase 3, *At3g63110*) (Table 3). In addition, genes involved in sulphate metabolism and in iron acquisition, transport and homeostasis were all up-regulated 2 hrs after increased N availability (see Additional file 5). Seven transcription factor genes were significantly induced or repressed after short-term N induction (2 hr) [see Additional file 5], including up-regulated Myb transcriptional factor genes (*At3g46130, At1g25550 *and *At1g68670*) and down-regulated *PAP1 *and *PAP2 *genes.

Table 5 summarizes some biological processes over-represented in genes responding to short- and long-term N availability increase (P < 0.01). When N was transiently increased, genes involved in transporter activities were up-regulated. Eleven of these genes, or 20% of the significant gene list (excluding unknown function genes), belong to the "transport" process according to GO. Genome-wide, there are 1398 member genes in this process, or close to 8% of the genome, indicating a clear increase in N stimulated nutrient transport activities (P-value = 0.002). Genes involved in nitrogen assimilation, carbon metabolism, sulfate assimilation and cell homeostasis were simultaneously up-regulated after a short-term N increase (Table 5). The up-regulated genes are over-represented in the process "response to cytokinin stimulus" after both the short- and longer-term N increase (Table 5). After the 24 hr N increase, 203 genes involved in primary metabolism, or 54.7% of the significant gene list, were up-regulated, and 121 genes involved in protein biosynthesis, or 32.61% of the list, were up-regulated (Table 5). Other processes with over-represented up-regulated genes include those involved in nitrogen compound biosynthesis, chlorophyll biosynthesis, organelle organization and biogenesis, DNA packaging, nucleosome assembly, ribonucleotide biosynthesis, translation, and the responses to auxin stimulus (Table 5). On the other hand, down-regulated genes are over-represented in processes such as flavonoid biosynthesis, phenylpropanoid biosynthesis, anthocyanin biosynthesis and starch biosynthesis (Table 5), reflecting the shift from secondary to primary metabolism when more N became available. Down-regulation of anthocyanin biosynthetic genes was also observed after the shorter-term N increase (Table 5). The expression of many genes was significantly altered only after longer-term N increase, suggesting some of the changes may result from a secondary response. The expression levels of many genes fluctuated, some quite dramatically, in response to different degrees of N stress or different lengths of N induction. The most striking change was found with the Myb factor *PAP2 (At1g66390)*, which was up-regulated over 3-fold under mild N stress, up-regulated 26-fold under severe N stress, down-regulated ~7-fold after a 2 hr N increase and continued to fall by over 16-fold after the 24 hr N increase (Table 3), indicating the close correlation between anthocyanin biosynthesis and N availability.

**Table 5:** Biological processes with significant genes over-represented after short- and long-term N availability increase

Biological Process	No. in Genome	% in Genome	2 hr N increase			24 hr N increase		
			
			No. in List	% in List	P-value	No. in List	% in List	P-value
**Up-regulated**								
transport	1398	7.7	11	20.4	0.002			
cell homeostasis	64	0.4	3	5.6	0.0009			
nitrate assimilation	7	0.04	2	3.7	0.0002			
sulfate assimilation	11	0.06	2	3.7	0.0005			
response to carbohydrate stimulus	29	0.16	2	3.7	0.003			
primary metabolism	5170	28.5				203	54.7	1.0E-26
protein biosynthesis	500	2.8				121	32.6	4.2E-98
organelle organization and biogenesis	439	2.4				45	12.1	3.5E-19
ribosome biogenesis and assembly	117	0.6				34	9.2	5.9E-30
translation	136	0.7				13	3.5	4.4E-06
response to auxin stimulus	191	1.1				12	3.2	0.0006
nucleosome assembly	56	0.3				9	2.4	1.8E-06
DNA packaging	106	0.6				9	2.4	0.0003
translational elongation	31	0.2				8	2.2	1.5E-07
nitrogen compound metabolism	124	0.7				8	2.2	0.004
amino acid biosynthesis	99	0.5				7	1.9	0.004
ribonucleotide biosynthesis	45	0.2				5	1.3	0.002
chlorophyll biosynthesis	14	0.08				4	1.1	0.0001
response to cytokinin stimulus	31	0.2	3	5.6	0.0001	4	1.1	0.003
**Down-regulated**								
response to abiotic stimulus	1091	6.0				40	15.5	3.2E-08
secondary metabolism	249	1.4				14	5.4	1.4E-05
response to abscisic acid stimulus	113	0.6				11	4.3	6.3E-07
phenylpropanoid biosynthesis	79	0.4				7	2.7	0.0001
flavonoid biosynthesis	30	0.2				5	1.9	6.0E-05
anthocyanin biosynthesis	8	0.04	1	10	0.004	3	1.2	0.0002
starch biosynthesis	7	0.04				2	0.8	0.004

The gene list was imported into GeneSpring. The number of genes in each biological process either in a whole genome (No. in Genome), or in the list (No. in list), as well as their corresponding percentage and p-value was generated by GeneSpring.

### Comparison with published microarray data

Wang et al [19] published microarray data for roots and shoots of Arabidopsis seedlings grown in a "pure" hydroponic system for 10 d on ammonium and then supplied with 0.25 mM nitrate for 20 min. They found that a total of 76 genes were up-regulated and 2 genes were down-regulated using a cutoff value of 2-fold change in shoots. Scheible et al [20] revealed genes affected by N-induction after 30 min or 3 hr from N-starved seedlings. The induction patterns and the majority of the nitrate-induced genes of our study overlap with their findings [19,20]. Most of the nitrate assimilation genes were induced in a similar fashion, such as *NR1, NR2*, *NiR*, *UPM1 *and two of the six *FNR *genes [19]. However, high affinity nitrate transport gene (*NRT2.1*) was induced significantly from N-starved seedlings [19,20], but not in our experiments. This is likely due to the different system used. Rather than being N-starved prior to the addition of a high level of nitrate, plants used in the current study were grown on a constant low level of nitrate, and there was some storage of free nitrate in the plants (Figure 1b). Our data presented here were from samples taken in the middle of the day to minimize diurnal effects, as nitrate levels in the plants could still fluctuate at dawn and at night and thus cause some corresponding transcriptional changes [52].

Wang et al [19] identified 6 transcription factor genes in shoots at least 2-fold induced or repressed after 20 min induction. In N-starved seedlings, Scheible *et al*. [20] identified 37 and 42 transcription factor genes, with altered expression after 30 min or 3 hr N induction respectively, with at least a 3-fold change. We identified 7 transcription factor genes significantly induced or repressed after short-term N induction (2 hr), all of which overlap with Scheible's gene list [20].

Additionally, Scheible et al [20] discovered 55 transcription factor genes at least 3-fold induced or repressed in the N-starved seedlings. We identified 3 transcription factor genes significantly induced or repressed under mild N stress and 39 under severe N stress. However, ~70% of those genes (two of the three from mild N stress, and 27 of the 39 from severe N stress) do not overlap with Scheible's gene list [20], indicating that plants have a very different regulatory system to cope with N starvation versus chronic N stress. These transcription factor genes not reported previously were highlighted in the Additional Files 2 and 3.

Recently, Lian et al reported expression profiles of 10,422 genes at an early stage of low N stress in rice seedling [24]. They germinated and grew rice hydroponically with normal nutrient conditions until the emergence of the fourth leaf, then transferred the seedlings to a low N solution and harvested shoot and roots separately 20 min, 1 and 2 hr after the treatment. They found the down-regulation of genes involved in photosynthesis and energy metabolism as well as extensive cross-talk between the responses to low N stress and those to biotic and abiotic stresses. However, the genes involved in N uptake and assimilation remained unchanged, which is different from findings in our study, probably due to the fact that their low N treatment was not severe enough to cause immediate N shortage as plant vacuoles have a storage capacity for nitrogen [24].

### Identification of putative nitrogen regulatory elements

**Figure 2:**
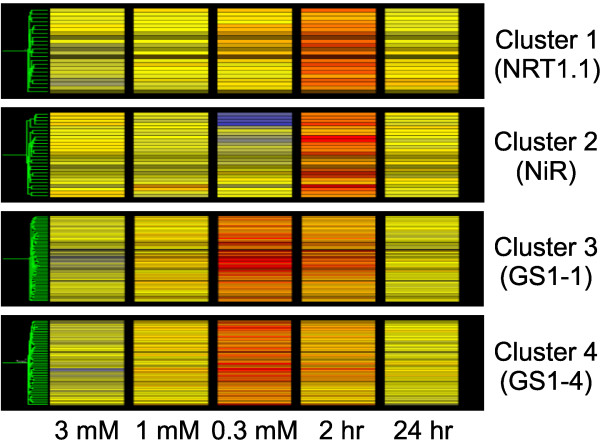
**Expression patterns of genes in each cluster**. Relative expression levels of genes in each cluster were presented. The warmer the colors are, the higher the expression levels are. 3 mM = the sufficient-N condition; 1 mM = the limiting-N condition; 0.3 mM = the stress-N condition; 2 hr = 2 hr induction; 24 hr = 24 hr induction. Each line represents one gene and the gene list is provided in Additional file 7.

The promoters of co-expressed genes are likely to share common regulatory motifs and are potentially regulated by a common set of transcription factors. Therefore, identification of *cis*-regulatory elements in the promoter regions becomes an important first step in uncovering new facets of transcriptional regulation networks for N responses. Target genes were selected from the three main stages of N assimilation, namely nitrate uptake, nitrate reduction and ammonium assimilation. Included were one of the major nitrate transporters (*NRT1.1*), nitrate reductase and nitrite reductase (*NR1, NR2 *and *NiR*), glutamine synthetase (*GS1-1 *and *GS1-4*) and asparagine synthetase (*AS2*). Data from the five N conditions (15 microarray chips) were used for clustering genes with the expression pattern most similar to those of the selected target genes. The cluster 1 (22 genes) included the target gene *NRT1.1*, as well as other transporter genes such as CLC-b chloride channel protein (*At3g27170*), the glutamine transporter (*At3g56200*), the sulphate transporter protein (*At1g23090*), the carbohydrate transporter (*At1g08930 *and *At4g17550*), and the sodium-dicarboxylate cotransporter (*At5g47560*), implying the coordinated regulation of different aspects of metabolism. The genes in cluster 1 had higher baseline expression levels under N stress. They were up-regulated after 2 hr N induction followed by an expression decline after 24 hrs (Figure 2). The cluster 2 (26 genes) included four target genes: *NR1, NR2, NiR *and *AS2*, as well as genes involved in glucose metabolism (*At1g24280*) and trehalose metabolism (*At5g51460*). Since C and N metabolism are very closely linked, it is interesting to note that some key enzymes in C and N metabolism fall into the same cluster. The genes in cluster 2 had a lower baseline under N stress. They were all up-regulated after 2 hr N induction and then decreased after 24 hrs (Figure 2). The cluster 3 (60 genes) included the target gene *GS1-1*. Genes involved in anthocyanin synthesis (*At4g22880*), and the rate-limiting enzyme in flavonol and anthocyanin biosynthesis chalcone synthase (*At5g13930*) were also in this cluster. The cluster 4 (50 genes) included the target gene *GS1-4*, as well as genes involved in anthocyanin synthesis (*At5g42800*), and the key enzyme in phenylprepanoid biosynthesis phenylalanine ammonia lyase (*At2g37040*), suggesting a close linkage between these metabolic pathways. The pattern of cluster 3 and 4 was similar in that they had higher baseline expression under N stress and they were repressed after the 2 hr N induction and then further decreased after 24 hrs (Figure 2). The entire list of genes in the four clusters is provided in Additional file 7, together with their functional and pathway assignments.

The upstream regions 1 kb onward from the ATG start sites of the genes present in the four clusters were analyzed for *cis *elements. A motif-module discovery algorithm, CisModule [53], was used to detect motif patterns and the 5 most highly over-represented patterns were found (Table 6). Those candidate motifs were subsequently searched against the PLACE database [54] to identify those containing previously published plant *cis *elements in both forward and reverse strand. GATA motifs were found in cluster 1, 2 and 4, Dof motifs were found in all four clusters, Myb motifs found in cluster 1 and 2, and WRKY and CCAAT motifs found in cluster 4 (Table 6).

**Table 6:** Motifs identified by CisModule and BioProspector

cluster	motifs	Known TF binding sequence contained (Reverse)	Binding (Reverse)
**CisModule**			
*Cluster 1 (NRT1.1)*	ARARRARRAG	AAAG	Dof
	CWMGTGKSSC		
	YAWAWAWMWAWAA	TAACAAA (GATA)	Myb (GATA)
	SRSCCACCAC		
	ARAGRARRAG	AAAG	Dof
*Cluster 2 (NiR)*	MAMAAAHAWAWA		
	CAYATCYMYCWC	(GATA, GGATA)	(GATA, Myb)
	GRRAGARRRRRR	AAAG	Dof
	ASAWRTATATR		
	RRAGARRARRRG	AAAG	Dof
*Cluster 3 (GS1-1)*	AMMAAAMAAAAAAA		
	RRARRARRAGA	AAAG	Dof
	YGRHSACGTSR		
	WDMTATATRWW		
	YGGHSACGTSR		
*Cluster 4 (GS1-4)*	TWKGTTTKGKT		
	CGTGRYYGSTVS	TGAC (CCAAT)	WRKY (CCAAT)
	KATATAKAKAT	GATA	GATA
	AGAARAMRAAR	AAAG	Dof
	GRSMCRYGWSR		

**BioProspector**			

*Cluster 1 (NRT1.1)*	MAAMARAMAAAAAAAA	AAAG	Dof
*Cluster 2 (NiR)*	MWTRGMCAWTCMTWWY	CCAAT	CCAAT
	RATAKGAATGTCYAWK	CCAAT	
	RAWTTGRTMGGAWTK	GATA (CCAAT)	GATA (CCAAT)
	TKTTTYTYTTTTTTTT	(AAAG)	(Dof)
	WTRGAYATTCMTATY	GATA	GATA
		GGATA	Myb
*Cluster 3 (GS1-1)*	GGWSACGTGGMRA		
	AARWAAAAAAAWAAAA		
*Cluster 4 (GS1-4)*	WWWTWTTRTTTTMTT		
	GARACAGASAGWKWSA	GATA (TAACTG)	GATA (Myb)

Candidate motifs from each cluster were identified by CisModule or BioProspector. They were subsequently searched against PLACE database to identify those containing previously published plant *cis *elements in the forward and reverse strand. Nucleotide abbreviations: R: A or G; Y: C or T; W: A or T; S: G or C; M: A or C; K: G or T; H: A, C or T; B: G, C or T; V: G, A or C; D: G, A or T; N: G, A, C or T.

To compare the results obtained from CisModule, another algorithm BioProspector was used to look for regulatory sequence motifs [55]. The top 5 over-represented patterns found are listed in Table 6. Various motif finding software programs typically yield candidate *cis *elements not entirely consistent with each other due to the differences in their underlying statistical algorithms. CisModule is based on a Bayesian hierarchical mixture model to infer motifs by their sequence background. BioProspector, on the other hand, uses a Gibbs sampling strategy to identify motifs. Less than five significant patterns were found for cluster 1, 3 and 4 by BioProspector, based on its significance level judged by its probability (p-value < 10^-5 ^in this case). These candidate motifs were subsequently searched against the PLACE database [54]. Again, GATA motifs were found in both cluster 2 and 4, Dof motifs in clusters 1 and 2, and Myb in cluster 2 (Table 6).

GATA motifs have been identified in the regulatory regions of many genes involved in nitrate assimilation such as nitrate reductase, nitrite reductase and glutamine synthetase [29,56,57]. Previously we identified regions of the spinach nitrite reductase (*NiR*) promoter that are involved in N regulation [28,29,58]. Footprinting assay suggests that GATA factors play a role in *NiR *gene regulation [29]. Therefore, the presence of GATA enriched motifs discovered by both algorithms in a NiR gene cluster lends substantial support to the validity of these putative motifs. Interestingly, the GATA motif was found in the NiR cluster as well as in the GS1-4 cluster by both algorithms. However, the expression patterns of these two clusters are very different, suggesting there are positive and negative GATA regulators. Also, the GATA motif was found by both algorithms only in the GS1-4 cluster, but not in the GS1-1 cluster, although the expression patterns of these two clusters are very similar, suggesting a complex regulatory system. Arabidopsis has 30 GATA transcription factor genes [39,59], but only a few of them have been functionally characterized [60-63], and none has been shown to be directly involved in N regulation.

The Dof transcription factors belong to the same C_2_C_2 _zinc finger transcription factor family as the GATA factors [64,65]. They are associated with expression of multiple genes involved in carbon metabolism in maize [66], but not in nitrogen metabolism. Dof factors were identified in all four clusters by CisModule and in two clusters by BioProspector. It is tempting to speculate that Dof factors could play an important role in nitrogen regulation. This speculation is supported by the fact that improved nitrogen assimilation and growth under low-nitrogen conditions could be achieved in Dof1 over-expressed plants [67].

In addition to the Dof factors, binding sequences for other factors such as CCAAT, Myb and WRKY were found in these clusters either by one of the algorithms or by both. Members of the CCAAT, Myb and WRKY factors have been shown to be involved in various abiotic stress regulation/tolerance [46,49-51,68]. Whether some of them are more specifically involved in N regulation requires further investigation. The putative regulatory elements identified in these clusters along with several other known motifs involved in N and C metabolism as well as stress response revealed a complex picture of plant N regulation.

## Conclusion

We used an Arabidopsis whole genome array for a global evaluation of gene expression under different N conditions. The differentially expressed genes identified provide additional insights into the coordination of the complex N responses of plants and the components of the N response mechanism. Putative N regulatory elements were identified along with several previously known motifs involved in N, C, and stress responses. A better understanding of the complex regulatory network for plant N responses will ultimately lead to strategies to improve N use efficiency in crop plants. Much remains to be done in order to fully construct the regulatory networks underlying this critical aspect of plant biology.

## Methods

### Microarray data availability

All expression data were collected in compliance with the MIAME standards [see Additional file 8] and are available through NASCArrays database with reference number NASCARRAYS-408.

### Plant growth conditions

Wild type *Arabidopsis thaliana *(Columbia ecotype) plants were grown under hydroponic conditions. Plant seeds were sown in rockwool cubes (25 × 25 × 40 mm; Fibrex Insulations Inc, ON, Canada), which were equilibrated with a balanced nutrient solution containing 10 mM KH_2_PO_4_, 2 mM MgSO_4_, 1 mM CaCl_2_, 0.1 mM Fe-EDTA, 50 μM H_3_BO_4_, 12 μM MnSO_4_, 1 μM ZnCl_2_, 1 μM CuSO_4_, 0.2 μM Na_2_MoO_4 _and varying levels of nitrate. Around 200 rockwool cubes were then placed in a tray which was connected to an 18-liter nutrient solution reservoir for sub-irrigation. Nutrient solution was pumped once a day to the tray from the reservoir to wet the rockwool completely and flowed back to the reservoir by an adjustable pump (Aquarium systems, OH, USA). Each of the above described hydroponic systems was one experimental unit used for the different nitrate levels. The electrical conductivity (EC) and pH levels of the nutrient solutions in the reservoir were monitored by TDSTestr 10 (OAKTON Instruments, IL, USA) and pH Pro Meter (Spectrum Technologies Inc. IL, USA) and adjusted to their target levels twice a week. There were three nitrate levels (treatments) in the experiment with the 3 mM nitrate as sufficient, 1 mM nitrate as the mild N-limiting condition and 0.3 mM nitrate as the severe N-limiting condition. The targeted EC was 670, 770, and 840 ppm respectively, and pH was around 6.0. Nitrate levels of the nutrient solutions in the reservoir were monitored by Cardy NO_3 _Nitrate Meter (Spectrum Technologies Inc. IL, USA). For the N induction, rockwool cubes with plants grown under 0.3 mM N were transferred to the tray with a connection to the 3 mM N reservoir. Nutrient solution was then pumped to the tray from each unit at 11 AM and shoots were harvested at 1 PM. The method to detect nitrate levels in the plants was described in Peng et al [69]. Plants were grown in a growth room with 16 hr lighting per day under fluorescent lamps (with a photosynthetically active radiation of 150 μmolm^-2^s^-1^) at 23°C and 8 hr dark at 18°C for three weeks before shoots were harvested. Shoot biomass was taken from an average of 6 to 8 plants and nitrate concentration from an average of 3 samples (each sample from a pool of 3 plants). In order to harvest sufficient material for RNA extraction, the number of plants pooled to run the microarray experiment was different. It was approximately 8, 10, and 16 plants respectively for the N-sufficient, mild N-stress and severe N-stress conditions. Plants used for RNA extraction were different from those for fresh weight and nitrate concentration, but they were grown under same conditions.

### Microarray hybridization

Five μg of total RNA from each sample was used to synthesize double-stranded cDNAs. Labeled complementary RNA, synthesized from the cDNA was hybridized to a custom designed Arabidopsis whole genome exon GeneChip array (SYNG002) as previously described [70]. The hybridization signals of the arrays were acquired by the GeneChip scanner 3000 and quantified by MAS 5.0 (Affymetrix). Each probe set measurement was summarized as a value of weighted average of all probes in a set, subtracting bottom 5% of average intensity of the entire array using a custom algorithm. The overall intensities of all probe sets of each array were further scaled to a target intensity of 100 to enable direct comparison.

### Microarray data analysis

Out of a total of 26412 × 15 = 396180 data points in the five treatment groups, the custom algorithm flagged 20149 or 5% as 'Absent' (A), 153373 or 39% as 'Marginal' (M), and 222658 or 56% as 'Present' (P). Stepwise gene filtering was conducted in GeneSpring (Agilent, CA, USA). First, for each of the five treatment groups, each gene must have either a 'P' or 'M' flag in all 3 replicate samples. This was followed by a filtering second step requiring that at least one of the three samples had a 'P' flag. This essentially guaranteed that every gene remaining in a group would be 'PMM', 'PPM', or 'PPP' among the three replicates. For pairwise group comparisons determining differentially expressed genes, genes common to both groups were identified and data exported, log_2 _transformed, and analyzed in Statistical Analysis of Microarray (SAM) as two class unpaired t-test and at a permutation number of 500 [35].

### Clustering analysis

For clustering analysis, those genes present in at least 2 out of the 5 treatment groups, totaling 17138, were exported into Gene Cluster 3.0 with the following clustering parameters selected: log2 transformation, Hierachical clustering, Correlation (centered), and Average linkage.

### Arabidopsis motif analyses

Gene clusters containing several target genes were identified with correlation coefficients within a cluster greater than 0.9. The corresponding 1 kb upstream sequences for each gene were downloaded from The Arabidopsis Information Resource [39] as of June 14, 2006 and used as input files for motif search in BioProspector [55] and CisModule [53]. For BioProspector, the entire 1 kb upstream sequence of Arabidopsis genome was used as the background distribution. The other parameters were set as follows: motif width, 6 to 16 bps; number of Monte Carlo simulations, 100; number of times to search for motifs, 100; number of top motifs to report, 10. For CisModule, all upstream sequences were masked by Repeatmasker [71] prior to motif analysis. The program settings were: number of motifs, 3 or 5; degenerate to independent motif sampler; total number of iterations, 1000; search both strands; and run dataset 10 times. Motif outputs from both BioProspector and CisModule were subsequently searched against PLACE database [54] as of August 31, 2006 to identify those containing previously published plant cis-elements.

## Authors' contributions

YMB designed and conducted the experiment, analyzed data and drafted the manuscript. RLW performed data analysis, searched putative regulatory elements and provided critical revisions to the manuscript. TZ coordinated the microarray experiments and provided insights on the motif search. SJR designed and coordinated the experiment and finalized the manuscript. All authors read and approved the final manuscript.

## Supplementary Material

Additional file 1 Validation of microarray results by semi-quantitative RT-PCR. Click here for file

Additional file 2 The significant gene list under mild N stress. Click here for file

Additional file 3 The significant gene list under severe N stress. Click here for file

Additional file 4 The overlapping gene list between mild and severe N stress. Click here for file

Additional file 5 The significant gene list after short-term N increase. Click here for file

Additional file 6 The significant gene list after long-term N increase. Click here for file

Additional file 7 The gene list in each cluster. Click here for file

Additional file 8 MIAME checklist. Click here for file
