# Supplementary figures and images for: Global transcription profiling reveals differential responses to chronic nitrogen stress and putative nitrogen regulatory components in Arabidopsis
Source: BMC Genomics. 2007 Aug 16;8:281. doi: 10.1186/1471-2164-8-281 (PMC1994689; doi:10.1186/1471-2164-8-281)

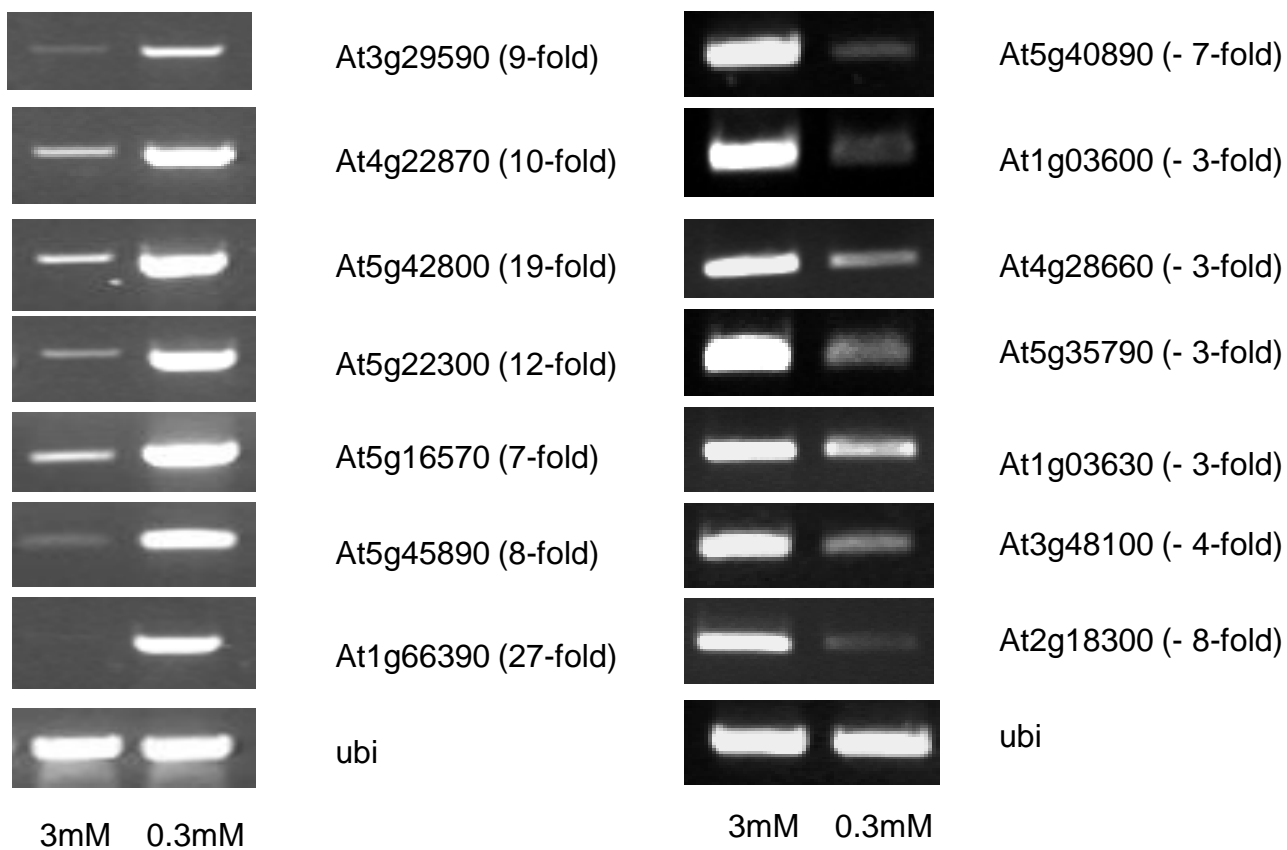

Note: Fold changes from microarray analysis were shown in brackets.

Supplement: Additional file 1 — Validation of microarray results by semi-quantitative RT-PCR. [file 1471-2164-8-281-S1.pdf]
