# Supplementary material for: Global transcription profiling reveals differential responses to chronic nitrogen stress and putative nitrogen regulatory components in Arabidopsis
Source: BMC Genomics. 2007 Aug 16;8:281. doi: 10.1186/1471-2164-8-281 (PMC1994689; doi:10.1186/1471-2164-8-281)
Supplement: Additional file 8 — MIAME checklist. [file 1471-2164-8-281-S8.doc]

MIAME checklist for Arabidopsis nitrogen (N) study

Experiment design:

- The goal of the experiment:

The goal of this study is to identify significantly differentially expressed genes under different degree of N stress or after different time frame of N induction as well as putative N regulatory elements.

- Experimental factors:

1. N concentrations (different degree of N stress)
2. N induction times (different time frame of N induction)
   - Experimental design:

Plants were grown under three N conditions: N-sufficient (3 mM N), N-limiting (1 mM N), and N-stress (0.3 mM N) and harvested at 3-week-old.

Also, at 2 hrs or 24 hrs before the harvest, some of the plants grown under the N-stress condition (0.3 mM) were transferred to the N-sufficient condition (3 mM nitrate), and then collected at the same time as those grown under different stable N conditions.

Genes differentially expressed were identified between (1) the N-sufficient to the N-limiting samples (3 mM N to 1 mM N), (2) the N-sufficient to the N-stress samples (3 mM N to 0.3 mM N), (3) the N-stress (0.3 mM N) to the 2 hr N induction samples, and (4) the N-stress (0.3 mM N) to the 24 hr N induction samples.

- Quality control:

Each condition had three biological replicates.

- Links to the database accession numbers:

NASCARRAYS-408

Samples used, extract preparation and labelling:

- The origin of each biological samples

(1) 3-week-old *Arabidopsis* *thaliana* (Columbia ecotype) grown under the N-sufficient condition (3 mM), 3 biological replicates;

(2) 3-week-old *Arabidopsis* *thaliana* (Columbia ecotype) grown under the N-limiting condition (1 mM), 3 biological replicates;

(3) 3-week-old *Arabidopsis* *thaliana* (Columbia ecotype) grown under the N-stress condition (0.3 mM), 3 biological replicates;

(4) 3-week-old *Arabidopsis* *thaliana* (Columbia ecotype) grown under the N-stress condition (0.3 mM) transferred to the N-sufficient condition (3 mM nitrate) for 2 hrs, 3 biological replicates;

(5) 3-week-old *Arabidopsis* *thaliana* (Columbia ecotype) grown under the N-stress condition (0.3 mM) transferred to the N-sufficient condition (3 mM nitrate) for 24 hrs, 3 biological replicates.

- Protocol for preparing the hybridization extract:

Total RNA was extracted using RNAwiz (Ambion), following manufacturers’ specifications.

- Labeling protocols:

labels used:
    - name (Biotin Labelled cRNA using Affymetrix Enzo Kit)
    - amount labelled (100 ug)
    - label method (In-vitro transcription method)

Hybridization procedures and parameters:

-   name (Affymetrix Antibody Amplification Protocol)
-   solution (Buffer: EDTA, NaCl, Tween20 according to Affymetrix
protocol)
-   blocking agent (Herring Sperm DNA (Promega) -> 100pg/ml.
Acetylated BSA (Sigma-Aldrich) -> 0.1mg/ml)
-   washing procedure
-   amount used (12.5 microgram Fragmented cRNA)
-   time (16 hours)
-   concentration (50 pg/ml)
-   volume (120 ul)
-   temperature (42^0^C)

Measurement data and specifications:

(1) scanning protocol
    - name (Affymetrix MAS 5.0 Standard Scanning)
    - scanner name (Affymetrix Scanner 3000)
    - software name (Affymetrix GCOS)
    - other details such as width, height, laserpower.
(2) image analysis protocol
-   name (Affymetrix MAS 5.0 Standard Image Analysis)
-   sofware name (Affymetrix GCOS)

Array design:

-   array type name (Affymetrix GeneChip oligonucleotide probe arrays)
-   array type platform (in situ)
-   array design name (Syngenta sySYNG002a Arabidopsis Genome Exon Array)
-   array design source (Affymetix)
-   array design element type (Synthetic oligo-nucleotides)
-   array design stranded (single)
